# Supplementary material for: Identification of photoperiod-induced specific miRNAs in the adrenal glands of Sunite sheep (Ovis aries)
Source: Front Vet Sci. 2022 Jul 22;9:888207. doi: 10.3389/fvets.2022.888207 (PMC9354845; doi:10.3389/fvets.2022.888207)
Supplement: Supplementary file 1 [file Data_Sheet_1.ZIP › Supplementary file/miRDeep2 file.zip!/miRDeep2 file/Know_Structure/SPLP42A1/oar-mir-194.pdf]

miRBase precursor : oar-mir-194
Total read count : 3930
oar-miR-194 read count : 3923
remaining reads : 7

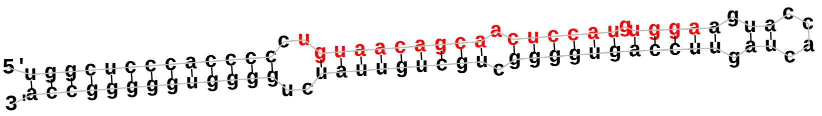

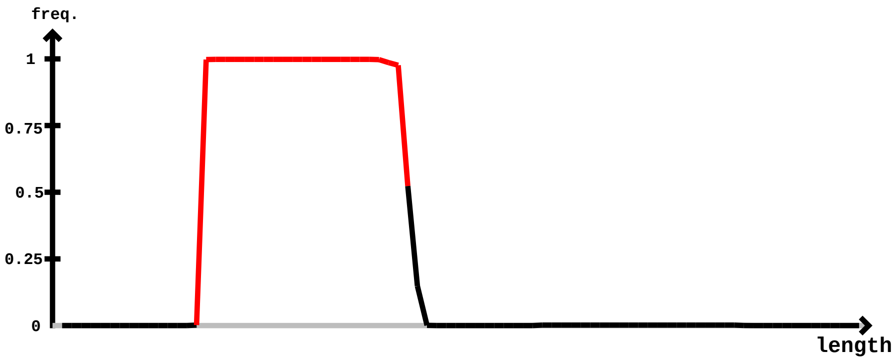

oar-miR-194

```
5'- uggcucccacccccuguaacagcaacuccaugugggaaguaccacuaguuccaguggggcugcuguuaucuggggugggggcca   -3'    exp
    ((((((((((((..(((((((((.((((((.(((((.((....)).))))))))))).)))))))))..))))))))))))     reads       mm          sample
    .............cuguaacagcaacuccaugu..................................................   2           0           seq
    .............cuguaacagcaacuccaugugg................................................   1           0           seq
    .............cuguaacagcaacuccaugugga...............................................   4           0           seq
    ..............uguaacagcaacuccaug...................................................   4           0           seq
    ..............uguaacagcaacuccaugu..................................................   42          0           seq
    ..............uguaacagcaacuccaugug.................................................   39          0           seq
    ..............uguaacagcaacuccaugugg................................................   1779        0           seq
    ..............uguaacagcaacuccaugugga...............................................   1468        0           seq
    ..............uguaacagcaacuccauguggaa..............................................   567         0           seq
    ..............uguaacagcaacuccauguggaC..............................................   11          1           seq
    ..............uguaacagcaacuccauguggaag.............................................   3           0           seq
    ...............guaacagcaacuccaugugga...............................................   1           0           seq
    ...............guaacagcaacuccauguggaa..............................................   2           0           seq
    .................................................ccaguggggcugcuguuaucu.............   6           0           seq
    .................................................ccaguggggcugcuguuaucug............   1           0           seq
```
